# DME-RWKV: An Interpretable Multimodal Deep Learning Framework for Predicting Anti-VEGF Response in Diabetic Macular Edema

**DOI:** 10.3390/bioengineering13010012

**Published:** 2025-12-24

**Authors:** Yan Liu, Xieyang Xu, Jiaying Zhang, Hui Wang, Ao Shen, Xuefei Song, Xiaofang Xu, Yao Fu

**Affiliations:** 1 Department of Ophthalmology, Ninth People’s Hospital, Shanghai Jiao Tong University School of Medicine, Shanghai 200011, China; liuyan1207@126.com (Y.L.); songxuefei@shsmu.edu.cn (X.S.); 2 Shanghai Key Laboratory of Orbital Diseases and Ocular Oncology, Shanghai 200011, China; 3 College of Information Science and Engineering, Hohai University, Changzhou 213200, China

**Keywords:** diabetic macular edema, epiretinal membrane, optical coherence tomography, ultra-widefield imaging, causal attention, curriculum learning, multimodal deep learning

## Abstract

Diabetic macular edema (DME) is a leading cause of vision loss, and predicting patients’ response to anti-vascular endothelial growth factor (anti-VEGF) therapy remains a clinical challenge. In this study, we developed an interpretable deep learning model for treatment prediction and biomarker analysis. We retrospectively analyzed 402 eyes from 371 patients with DME. The proposed DME-Receptance Weighted Key Value (RWKV) integrates optical coherence tomography (OCT) and ultra-widefield (UWF) imaging using Causal Attention Learning (CAL), curriculum learning, and global completion (GC) loss to enhance microlesion detection and structural consistency. The model achieved a Dice coefficient of 71.91 ± 8.50% for OCT biomarker segmentation and an AUC of 84.36% for predicting anti-VEGF response, outperforming state-of-the-art methods. By mimicking clinical reasoning with multimodal integration, DME-RWKV demonstrated strong interpretability and robustness, providing a promising AI framework for precise and explainable prediction of anti-VEGF treatment outcomes in DME.

## 1. Introduction

Diabetic macular edema (DME) is one of the leading causes of vision impairment among working-age individuals with diabetes, affecting approximately 6–7% of the diabetic population worldwide [1,2]. Intravitreal injection of anti-vascular endothelial growth factor (anti-VEGF) agents has become the first-line therapy for DME due to its proven ability to reduce macular swelling and improve visual acuity. However, treatment response varies markedly among patients: while some experience substantial anatomical and functional recovery, others show suboptimal or even refractory responses, leading to repeated injections, increased costs, and reduced compliance [3]. Predicting individual therapeutic efficacy before initiating therapy, especially when anti-VEGF treatment has failed, allows for timely switch to more effective alternative therapies, ultimately securing better visual prognosis for patients [4]. Therefore, predicting individual therapeutic outcomes prior to treatment initiation is crucial for guiding personalized management and optimizing healthcare resources.

Among known prognostic factors, the presence of an epiretinal membrane (ERM) has been recognized as a potential determinant of reduced anti-VEGF responsiveness as it can exert tangential traction on the macula, distort macular architecture, increase retinal thickness, and impede drug diffusion [5,6,7,8]. A study by Kakihara S et al. found that among DME patients undergoing anti-VEGF therapy, those with ERM received significantly more anti-VEGF injections during the follow-up period than those without ERM [5]. Therefore, these authors concluded that ERM is a marker of DR severity and an independent risk factor for increased demand for anti-VEGF therapy. An ERM indicates a more severe condition and a poorer response to anti-VEGF treatment [4,9,10]. For these patients, aggressive surgical intervention (vitrectomy combined with ERM peeling) is considered a key treatment option [6]. Multiple clinical studies have shown that DME patients with ERM often exhibit limited improvements in both best-corrected visual acuity and central macular thickness (CMT) after anti-VEGF therapy [11,12]. Mechanical traction problems at the vitreoretinal interface (such as vitreomacular traction and ERM) are key reasons for the poor response to anti-VEGF therapy in some DME patients, highlighting the importance of identifying these structural abnormalities before treatment to develop personalized treatment plans [12]. Conversely, ERM peeling—particularly when guided by intraoperative optical coherence tomography (iOCT) or performed with fovea-sparing techniques—has been demonstrated to enhance retinal anatomical restoration and visual recovery in diabetic patients [13,14]. These findings underscore the clinical importance of ERM as a key prognostic biomarker worthy of inclusion in efficacy prediction frameworks.

Notably, efforts to predict anti-VEGF outcomes based on ERM-related parameters predated the application of artificial intelligence (AI). Earlier approaches employed optical coherence tomography (OCT) for the qualitative grading or quantitative measurements of ERM, combined with clinical variables such as age, diabetes duration, baseline visual acuity, and CMT, to construct statistical prediction models [11,12]. The health of the microstructures (inner retinal layer and photoreceptors) revealed by OCT determines the potential for visual recovery. This helps guide clinical decisions and optimize surgical timing. The retinal microstructures observed through OCT, particularly the condition of the inner retinal layer and the integrity of the ellipsoidal zone (EZ), have significant predictive value for postoperative visual acuity [15]. However, manual feature-engineered methods suffer from inherent subjectivity and limited dimensionality, making it difficult to capture fine-grained microstructural abnormalities—such as intraretinal cysts (IRCs) and subretinal fluid (SRF) and their complex spatial interactions—thereby restricting predictive performance.

OCT, as the gold standard for DME assessment, provides high-resolution visualization of retinal microstructures, including ERM and associated pathological changes [16,17,18]. Traditional prediction methods, including regression tree models, have limited ability to capture the complex microstructural interactions in OCT and often rely on crude features such as baseline best-corrected visual acuity (BCVA) or CMT, resulting in poor predictive performance [3,19,20]. These findings collectively emphasize the need for a computational framework capable of modeling subtle OCT structural changes, spatial lesion patterns, and multimodal interactions to better predict treatment outcomes for DME with ERM. With the rapid evolution of deep learning, AI-based OCT analysis has achieved substantial advances in disease classification, segmentation, and prognosis prediction [21,22]. Convolutional neural networks (CNNs) excel at local feature extraction but have limited receptive fields, whereas vision transformers (ViTs) leverage self-attention mechanisms to capture global dependencies at the expense of quadratic computational cost [23]. To balance accuracy and efficiency, new architectures such as the Receptance Weighted Key Value (RWKV) model have emerged, integrating the parallel learning capacity of transformers with the inference efficiency of recurrent neural networks (RNNs), achieving linear computational complexity O(N) [24,25,26,27]. Its visual adaptation, Vision-RWKV (VRWKV), has demonstrated superior performance in global context modeling and efficiency in medical imaging tasks [28].

In clinical practice, the comprehensive assessment of DME not only relies on OCT, but also combines it with fundus photography and other examinations to improve the classification accuracy of ophthalmic diseases [29,30]. He X proposed a multimodal retinal image classification method based on a modality-specific attention network [29]. This technology has significant application potential for assisting in the diagnosis of various retinal diseases, such as DR and age-related macular degeneration (AMD). Yoo TK also explored the combination of OCT and fundus images, suggesting that a combined approach can improve the accuracy of deep learning-based diagnosis of AMD [30]. Zuo Q proposed a multi-resolution visual Mamba network that captures different levels of retinal features using a single modality based on OCT [31]. While capturing global information, it may overlook local details and complex spatial relationships. Beyond OCT, comprehensive evaluation of DME in clinical practice often incorporates ultra-widefield (UWF) imaging, which provides valuable information on peripheral retinal lesions, ischemic areas, prior laser scars, and other features that are typically invisible in OCT’s limited field of view [28,32]. Theoretically, integrating OCT and UWF imaging enables a multimodal representation of both central and peripheral retinal pathology, offering a more complete understanding of disease severity and potential treatment response. However, most existing predictive models have relied on a single imaging modality, and multimodal AI frameworks combining OCT and UWF data remain largely unexplored.

Building upon these observations, the present study proposes a novel VRWKV-based architecture that integrates ERM spatial features for simultaneous anti-VEGF response prediction and OCT biomarker segmentation. The model incorporates a Causal Attention Learning (CAL) module to identify causally relevant regions, a curriculum learning strategy to emulate the progressive reasoning process of clinicians, and a global completion (GC) loss function to enhance small-lesion continuity (termed DME-RWKV). Furthermore, a multimodal OCT + UWF input pathway is constructed to simulate a comprehensive clinical assessment, aiming to improve prediction accuracy and interpretability, and ultimately supporting more individualized DME treatment planning.

## 2. Materials and Methods

### 2.1. Study Population

This retrospective study included 402 eyes from 371 patients diagnosed with DME at the Ninth People’s Hospital between October 2023 and February 2025. All patients were divided into a responsive group and a non-responsive group based on changes in CMT on OCT before and after treatment. The patient group responding to anti-VEGF treatment was defined as those whose CMT decreased by at least 10% after treatment. The responsive group consisted of 147 patients, and the non-responsive group consisted of 224 patients.

The inclusion criteria were as follows: patients with DME who received anti-VEGF therapy and had follow-up examinations both before treatment and at three months after injection. The exclusion criteria included a history of intraocular surgery or ocular trauma, high myopia (spherical equivalent ≤ −6.0 D), hyperopia (spherical equivalent ≥ +2.0 D), other macular diseases, retinal vein occlusion, cataract, glaucoma, or uveitis, uncontrolled hypertension, and poor-quality OCT images.

Demographic and preoperative clinical data included patient age, gender, intraocular pressure (IOP), and CMT. ERM was annotated by two ophthalmologists based on OCT images and verified by a senior retinal specialist.

### 2.2. OCT and UWF Image Collection

All eyes in this study were imaged using the spectral-domain OCT (Spectralis OCT, Heidelberg Engineering, Heidelberg, Germany) at baseline and 3 months post-injection, and the scans were tracked. Scans with a signal strength index greater than 25 and without residual motion artifacts were retained for further analysis. For each patient, a single 9 mm horizontal scan passing through the fovea was selected from each follow-up visit. Optomap (Daytona, Optos, Dunfermline, UK) UWF images were captured with the macula centered.

### 2.3. DME-RWKV Network

**Figure 1:**
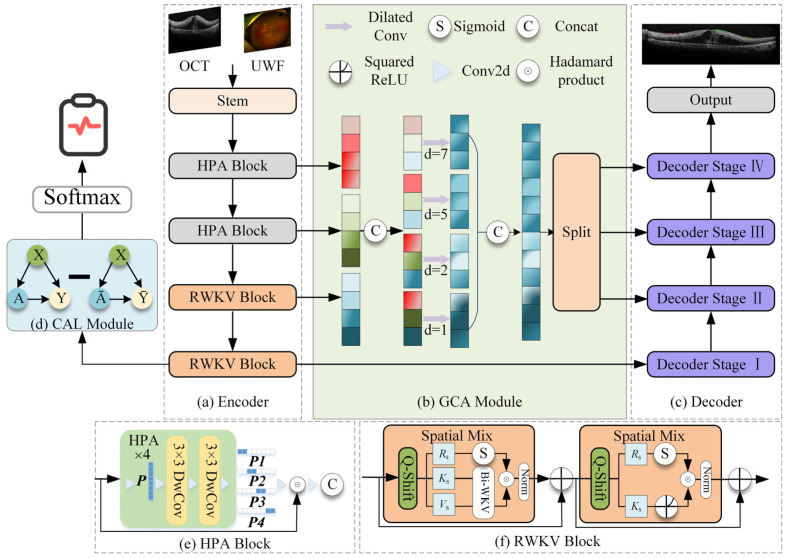
DME-RWKV network structure. The framework consists of four main components: (**a**) the encoder, which employs a hybrid design using (**e**) HPA Blocks to extract local spatial features and (**f**) RWKV Blocks to capture global long-range dependencies; (**b**) the GCA (Global Context Attention) module, which fuses multi-scale contextual information via dilated convolutions with varying dilation rates (*d* = 1, 2, 5, 7); (**c**) the decoder, which progressively recovers spatial resolution for the final segmentation output; and (**d**) the CAL module, which provides auxiliary supervision to enhance feature discriminability.

The proposed model adopts a classic encoder–GCA fusion module–decoder architecture, integrating RWKV-based efficient modeling modules and specifically designed attention mechanisms at each stage, aiming to efficiently process high-resolution OCT images while enhancing global understanding and local detail extraction capabilities (Figure 1).

#### 2.3.1. Encoder

The encoder employs a hybrid architecture designed to balance computational efficiency with feature extraction power. The first two layers consist of the Hadamard Product Attention (HPA) module. Based on a lightweight Hadamard convolution structure, this module replaces traditional heavy convolution or attention mechanisms to effectively aggregate local features. By utilizing rapid Hadamard transforms, the HPA module significantly reduces the network’s parameter count and computational burden, making it highly suitable for processing high-resolution OCT images.

Following this local feature aggregation, the HPA module works seamlessly with the subsequent VRWKV encoding blocks. Built upon the VRWKV architecture, these blocks utilize global receptive modules with linear complexity (e.g., Bi-WKV, SQ-Shift) to capture long-range dependencies and broad contextual information. This hybrid design allows the encoder to incorporate powerful global information while ensuring efficient feature extraction, ultimately forming a structure that balances precision with speed.

#### 2.3.2. GCA Fusion Module

The Global Context Attention (GCA) module is designed to integrate information from different encoder layers, balancing global structure and local anomaly information. It includes two mechanisms: global attention map generation and multi-scale fusion. Global attention map generation is used to generate soft attention maps based on the encoded features, guiding the subsequent decoder to focus on prognosis-related areas (such as IRC, SRF, and ERM). In addition, the GCA module performs multi-scale fusion by combining shallow high-resolution features with deeper semantic information, enhancing the model’s ability to recognize small lesions.

#### 2.3.3. Decoder

The decoder is structured into four distinct stages, symmetrically corresponding to the encoder’s hierarchical levels. This four-stage design is adopted to progressively recover spatial resolution from deep semantic features while mitigating the loss of fine details. In each stage, the decoder combines upsampled high-level semantics with corresponding low-level details from the encoder via skip connections. This stepwise reconstruction strategy allows the model to effectively bridge the semantic gap between encoder and decoder features, ensuring precise boundary delineation for small lesions.

The decoder produces two primary outputs: a region-of-interest (ROI) segmentation map, which automatically delineates key structures in OCT images (e.g., ERM, IRC, and SRF), and a therapeutic efficacy prediction vector, which integrates attention-weighted information to predict treatment response. During training, the loss function includes segmentation loss (such as Dice/BCE) and therapeutic prediction loss, incorporating a GC loss to further improve sensitivity to small lesions.

### 2.4. GCA Feature Fusion Module

The GCA feature fusion module is designed to efficiently and effectively integrate multi-scale features from different stages of the encoder. Traditional segmentation networks often use a “bridge” or bottleneck layer that simply concatenates or sums features of different resolutions, which tends to underutilize contextual information and increases unnecessary computational overhead. The proposed GCA module addresses these limitations by explicitly modeling long-range dependencies and selectively enhancing key informative features while suppressing redundant or noisy responses.

The GCA module is structured in three stages:

(1) Feature alignment: Lightweight upsampling or downsampling operations align multi-resolution features spatially, ensuring consistent feature map sizes during fusion.
(2) Global context modeling: By combining global average pooling and learnable attention weights, semantic representations are aggregated, enabling the network to retain important local details while capturing global scene information.
(3) Global and local fusion: The globally enhanced features are fused with high-resolution local features via element-wise multiplication (Hadamard fusion) and residual addition, preserving fine-grained structural details while obtaining rich contextual information.

Replacing traditional bridge stages with the GCA module offers two major advantages. The first is enhanced cross-scale interaction: multi-level features are adaptively weighted based on their global relevance, rather than being equally merged. The second is improved computational efficiency: redundant feature channels are suppressed, thereby reducing parameters and computational costs (FLOPs) without compromising prediction performance.

In the DME-RWKV network, the GCA module serves as a critical intermediate processing stage, providing balanced feature representation between the encoder and decoder. This design is particularly important in retinal OCT image analysis, where both fine pathological structures and broader retinal contextual information need to be considered.

### 2.5. CAL Module

We propose the first use of a CAL module before the prediction head in the RWKV-based encoder. The module consists of one linear layer, one ReLU layer, and another linear layer, which optimizes the quality of the attention map via causal inference, thereby guiding the network to focus on features directly related to the factual structure. Specifically, given input features X and a causal attention map A, the intervention operation do(·) generates a causal attention feature map $\bar{A}$, where the do(·) operation uniformly applies randomly generated weight maps of the same size as the attention maps. The network’s output prediction $Y_{\mathrm{effect}}$ is represented as the difference between the predicted values based on the results Y and the predictions based on the non-result attention maps $\hat{Y}$. The formula is as follows:

$$Y_{\mathrm{effect}}\;={\;E}_{A\sim \gamma }[Y(A\;=\;A,\;X\;=\;X)–Y(\mathrm{do}(A\;=\;\bar{A}),\;X\;=\;X)],$$

where γ is the distribution of causal attention, and Y represents the prediction head. The final prediction $Y_{\mathrm{effect}}$ is the output after passing through a Softmax layer.

### 2.6. Curriculum Learning

**Figure 2:**
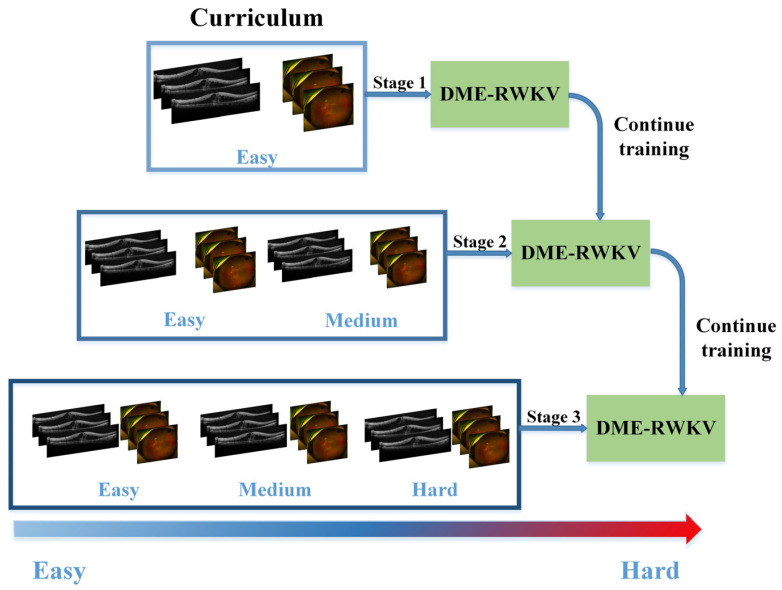
The flowchart of curriculum learning.

In terms of visual learning, we integrate various difficulty levels of diagnosing DME therapeutic efficacy from UWF and OCT images, taking into account factors such as signal intensity, area size, and image quality within the ROI. The training curriculum for the DME-RWKV network is designed for three levels of difficulty: easy, medium, and hard. This approach allows the network to first learn features associated with clear and easily predictable treatment responses, followed by progressively harder cases, thus enhancing the overall training process (Figure 2).

### 2.7. GC Loss

We observed that certain lesion areas in OCT images are very small or elongated, presenting significant challenges for accurate segmentation. Traditional methods like Dice Loss focus primarily on pixel-wise overlap; consequently, for these fine structures, even a few incorrect pixels can cause topological “breaks” or fragmentation. To address this, we propose a GC loss function designed to maintain the topological structural integrity of lesion regions.

**Figure 3:**
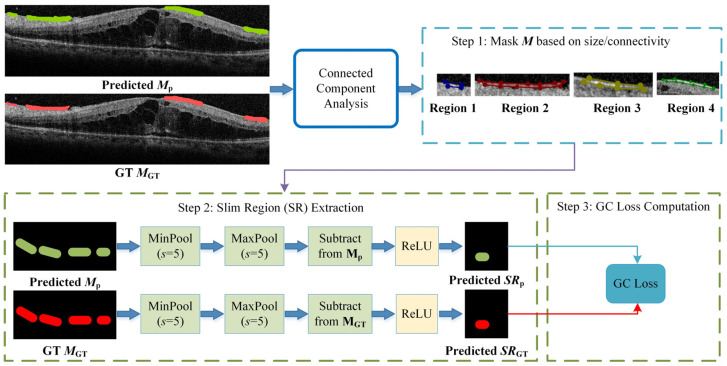
The algorithmic flowchart of the GC loss calculation. The process involves three stages: Step 1: filtering lesion masks using connected-component analysis to identify target regions; Step 2: extracting the topological skeletons (Slim Regions, SRs) from both prediction and GT using MinPool and MaxPool operations; and Step 3: computing the final GC loss based on the structural difference between the extracted skeletons.

**Figure 4:**
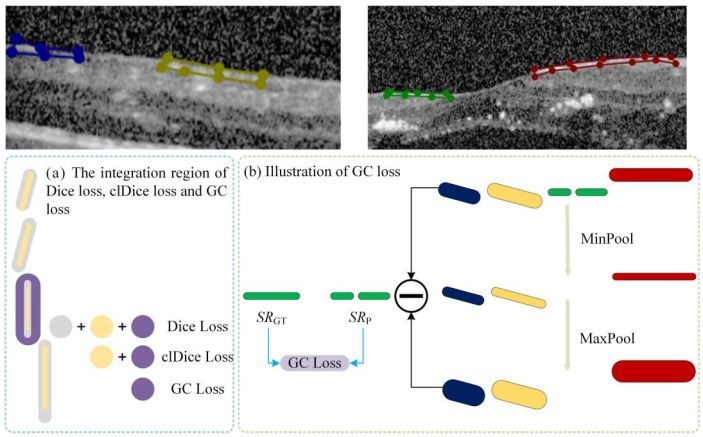
Schematic illustration of the GC loss function. The original semantic mask is decomposed into ***N*** independent instances using connected component analysis. Different colors represent distinct label values assigned to each connected region.

The specific calculation workflow of the GC loss function is illustrated in Figure 3. The process consists of three key steps: First (Step 1 in Figure 3), a connected-component analysis is applied to both the predicted map (***M***_p_) and the ground truth (***M***_GT_) to isolate specific lesion regions based on size and connectivity, ensuring the loss function focuses on relevant micro-structures. Second (Step 2 in Figure 4), the method extracts the skeletal structures, termed Slim Regions (SRs), from these masked areas. The core mechanism of this extraction is conceptually illustrated in Figure 4. It utilizes a morphological opening-like operation involving MinPool and MaxPool layers to capture the central topological line. The mathematical formulation is as follows:

*SR* = *ReLU*(*M–maxpool* (*minpool*(*M*,*s*),*s*)),

where *M* represents the lesion mask, and *s* is the kernel size of the pooling operations. In this study, *s* is set to 5 to efficiently extract the central line representing the lesion’s basic topology. Finally, (Step 3 in Figure 3), the GC loss is computed by minimizing the difference between the extracted skeletons of the prediction *SR*_p_ and the ground truth *SR*_GT_, thereby enforcing structural continuity in the final segmentation.

## 3. Results

### 3.1. Patient Characteristics

The demographic characteristics of the study cohorts are shown in Table 1. The study cohort comprised a total of 371 patients. The mean age of the participants was 63.54 ± 12.01 years. Regarding gender distribution, the majority of patients were male (218, 58.76%), while female patients accounted for 153 cases (41.24%). No significant differences were found between the responsive and non-responsive groups in terms of ERM percentage (*p* = 0.137) and IOP (*p* = 0.726). The CMT of the responsive group was higher (530.76 ± 230.57) than that of the non-responsive group (361.33 ± 184.76). The BCVA of the responsive group was also worse (0.19 ± 0.18).

### 3.2. Experiment Details

**Figure 5:**
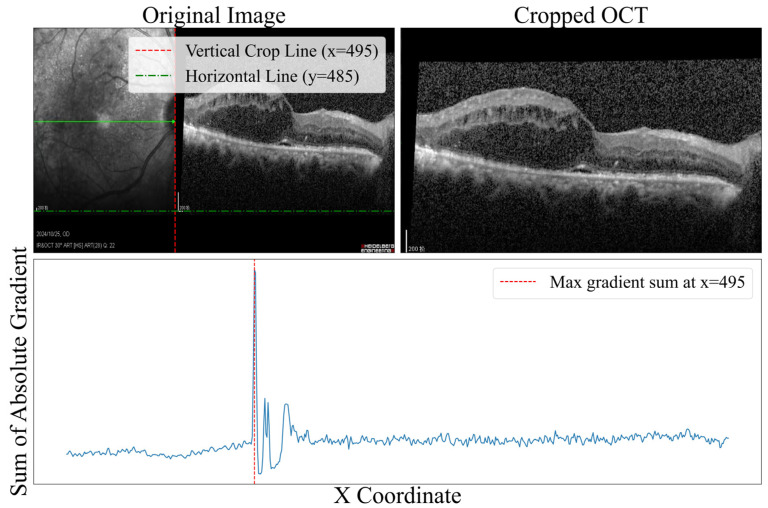
Image boundary detection based on the horizontal gradient of the original OCT image. The blue line represents the magnitude of gradient change in the horizontal direction, where the peak indicates the optimal crop position.

Prior to model training, the raw data underwent a series of preprocessing steps. First, a B-scan region was cropped from the raw OCT images as the network input. This process computed the gradient change in the horizontal direction of the original OCT image, as shown in Figure 5; when the gradient was maximized, the image boundary was defined, and the B-scan region was cropped accordingly. Subsequently, the images were resized to 512 × 512 × 3 to ensure uniform input dimensions. Lastly, to minimize pixel intensity variations across images from different patients, all inputs were normalized using Z-Score normalization.

Following preprocessing, the dataset—comprising 402 eyes with paired OCT and UWF images—was organized for training and evaluation. We randomly partitioned the dataset into a training set and an independent testing set at a 4:1 ratio, ensuring no data leakage. This resulted in 321 eyes allocated for training and 81 eyes reserved for testing. To ensure robust hyperparameter optimization, we employed a 5-fold cross-validation strategy on the training set. The validation results from the five folds were aggregated to assess performance, and the model achieving the highest validation accuracy was subsequently evaluated on the unseen testing set to report the final results.

### 3.3. Experimental Content

#### 3.3.1. Impact of Multimodal Data Fusion

To validate the specific contribution of integrating ultra-widefield (UWF) imaging with OCT, we conducted a quantitative ablation study comparing the model’s performance under single-modal (OCT-only and UWF-only) and multimodal (OCT + UWF) configurations. The results are presented in Table 2. Note that since the segmentation task targeted specific lesions within the OCT B-scans, the UWF modality alone could not perform this task and was evaluated solely on classification metrics. As shown in Table 2, the OCT-only baseline achieved a Dice coefficient of 62.26% for segmentation and an AUC of 75.26% for efficacy prediction. While the UWF-only input demonstrated comparable diagnostic capability (AUC 75.99%) to OCT alone, it lacked the depth information required for structural segmentation.

However, the proposed multimodal (OCT + UWF) fusion yielded the most superior performance across all metrics. For the prediction task, the integration of UWF boosted the AUC to 79.69% and accuracy to 79.61%, significantly outperforming both single-modal baselines. Remarkably, this fusion also enhanced the OCT segmentation task, increasing the Dice coefficient from 62.26% to 67.32%. This suggests that the peripheral retinal context provided by UWF helps the shared encoder learn more robust and discriminative feature representations, which in turn benefits the localization of micro-lesions in OCT images. These results confirm that fusing global peripheral information (UWF) with local cross-sectional details (OCT) is essential for maximizing both diagnostic precision and segmentation accuracy.

#### 3.3.2. Performance Comparison of Each Module in DME-RWKV

To comprehensively evaluate the performance of the DME-RWKV model, we conducted a series of detailed ablation studies to validate the effectiveness of each key module. As presented in Table 3 and Table 4, the complete model, which integrates the HPA, CAL, and GCA modules, demonstrated the most superior performance across both the segmentation and classification tasks. In the segmentation task, the complete model achieved a Dice coefficient of 67.32 ± 8.35%, an IOU of 51.33 ± 9.73, and a 95% HD of 11.91 ± 4.27 mm, outperforming all other module combinations.

**Figure 6:**
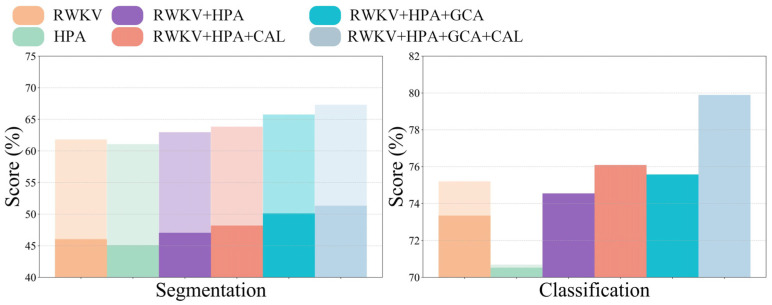
Performance comparison across different modules.

Similarly, in the classification task, the complete model outperformed on the three key metrics, with an area under the curve (AUC) of 79.89%, an accuracy (ACC) of 79.61%, and a sensitivity (SEN) of 83.41%. Figure 6 visually illustrates the significant stepwise performance increase as the HPA, CAL, and GCA modules are progressively integrated, strongly confirming the positive contribution of each component to the overall performance.

**Figure 7:**
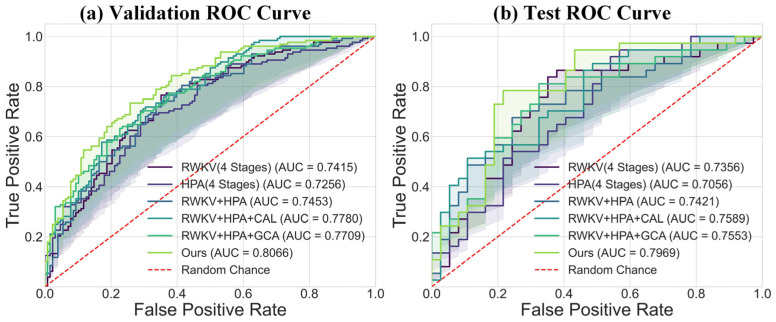
Comparison of ROC curves between the proposed model (“Ours”) and baseline models: (**a**) ROC curve comparison on the validation set. The results show that our model (AUC = 0.8056) outperforms all compared models, including RWKV (AUC = 0.7415), HPA (AUC = 0.7256), and their combined variants. (**b**) ROC curve comparison on the test set. Our model (AUC = 0.7959) also achieves the best performance on the test set, confirming its superior generalization ability.

**Figure 8:**
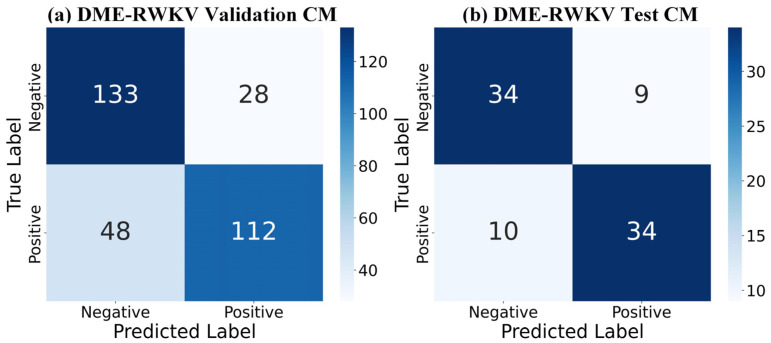
Confusion matrix (CM) for the DME-RWKV model: (**a**) Performance on the validation set. (**b**) Performance on the test set. Both matrices show identical results: 95 true negatives (TNs), 33 false positives (FPs), 34 false negatives (FNs), and 94 true positives (TPs).

Regarding the overall classification efficacy, the receiver operating characteristic (ROC) curves in Figure 7 further substantiate the superiority of our model. The complete model achieved AUC values of 0.7969 and 0.7996 on the validation and test sets, respectively, which are markedly better than those of other variants, demonstrating its strong discriminative ability and good generalization performance. The confusion matrix comparison in Figure 8 precisely illustrates the model’s prediction distribution for positive and negative samples. In summary, our experimental results consistently and robustly validate the effectiveness of the proposed DME-RWKV architecture and confirm the synergistic effect among its constituent modules.

#### 3.3.3. Performance Comparison of Joint Diagnosis and Segmentation in DME-RWKV

To validate the efficacy of the joint learning framework, we compared the model’s performance under both single-task and joint-task training regimes. As shown in Table 5, joint training significantly boosted the performance of both sub-tasks. Specifically, the Dice coefficient and IOU for the segmentation task improved from 65.36% and 49.45% to 67.32% and 51.33%, respectively. At the same time, the performance gain for the prediction task was even more substantial, with its key metrics—AUC, ACC, and SEN—increasing markedly from 77.23%, 74.69%, and 69.87% to 79.89%, 79.61%, and 83.41%, respectively. These results strongly demonstrate a mutually synergistic effect between the segmentation and prediction tasks, confirming that the strategy of joint optimization via shared feature representations is highly effective in enhancing the model’s overall comprehensive performance.

#### 3.3.4. Diagnosis and Segmentation Performance Comparison of DME-RWKV After Incorporating Curriculum Learning

We further evaluated the impact of different loss function configurations and the key design parameter *s* (target branch width) within the GC loss, as detailed in Table 6. While incorporating the clDice loss enhanced segmentation specificity (increasing to 85.12%), it significantly compromised sensitivity (dropping to 72.06%), indicating a bias toward conservative predictions. In contrast, the combination of DCE and GC loss achieved an optimal overall performance, particularly with $s$ set to 5. This configuration yielded the highest AUC (81.98%) and Dice coefficient (70.69%), maintaining a superior balance between sensitivity (74.70%) and specificity (82.24%). Sensitivity analysis of the parameter *s* further confirmed that *s* = 5 is the most robust setting for our dataset; reducing the width to *s* = 3 led to the lowest diagnostic performance (AUC of 77.01%), likely due to noise susceptibility, while increasing it to *s* = 7 resulted in a slight degradation in segmentation accuracy (Dice coefficient of 69.99%).

#### 3.3.5. Comparison of Diagnostic Performance with Other SOTA Methods

**Figure 9:**
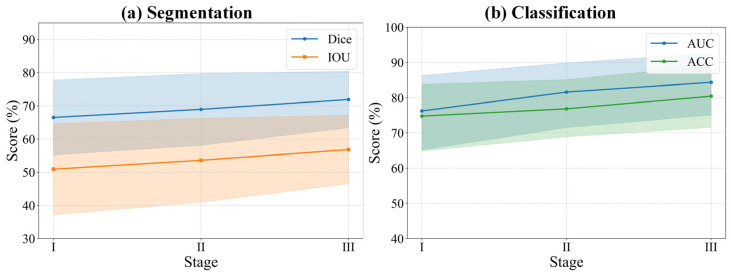
Relationship between segmentation and classification performance and curriculum learning. The solid lines represent the mean performance scores across different stages. The shaded areas indicate the 95% confidence intervals, representing the upper and lower bounds of the standard deviation.

To evaluate the effectiveness of our introduced curriculum learning strategy, we conducted a quantitative analysis of the model’s performance evolution across three learning phases (Stages I, II, and III). As presented in Table 7 and Figure 9, the experimental results clearly indicate a sustained and significant improvement in both segmentation and classification performance as the curriculum progressed from easy to difficult. Specifically, from Stage I to Stage III, the model’s segmentation performance steadily enhanced, with the Dice coefficient increasing from 66.47% to 71.91% and the IOU rising from 50.90% to 56.81%. Concurrently, the classification (diagnostic) ability also achieved a qualitative leap, with the AUC sharply increasing from 76.22% to 84.36% and accuracy improving from 74.75% to 80.44%. Notably, specificity reached a peak of 84.49% in Stage III, indicating that the model became exceptionally proficient at distinguishing negative samples during the later learning phases. The trend lines in Figure 9 visually demonstrate this progressive performance enhancement process. This series of results powerfully substantiates the effectiveness of the curriculum learning strategy. By guiding the model to first learn general features from easier samples, then gradually transitioning to the distinctive features of complex samples, this strategy effectively mitigated training difficulty and enabled the model to construct more robust and generalizable feature representations, ultimately leading to superior comprehensive performance.

#### 3.3.6. Comparison of Segmentation Performance with Other SOTA Methods

**Figure 10:**
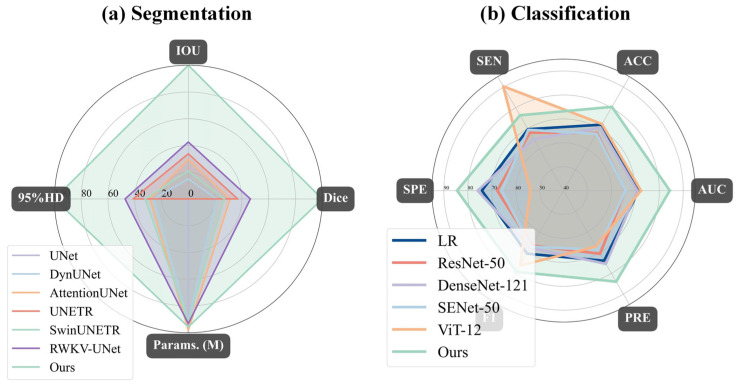
Radar chart comparison of the proposed model (“Ours”) against baseline models for (**a**) segmentation and (**b**) classification tasks: (**a**) For segmentation, our model (light green) achieves state-of-the-art performance, demonstrating the highest IOU and Dice scores while simultaneously having the lowest 95% Hausdorff Distance (95%HD). (**b**) For classification, our model again outperforms all baseline models (including ResNet-50, DenseNet-121, and ViT-12) by achieving the highest scores across all five metrics: specificity (SPE), accuracy (ACC), AUC, precision (PRE), and F1-score.

**Figure 11:**
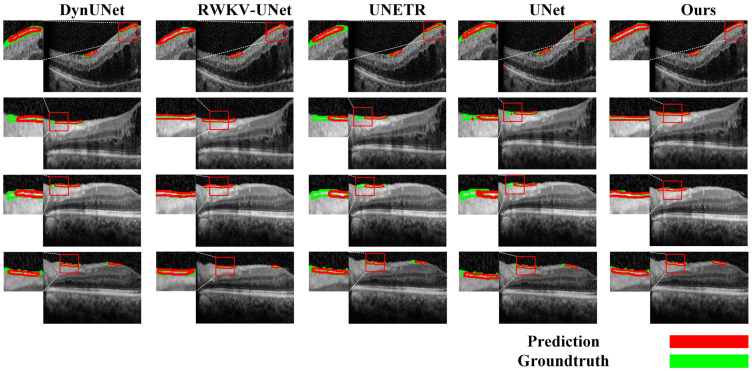
Qualitative evaluation of the segmentation task.

To comprehensively validate the superiority of our proposed model, we conducted a detailed performance comparison against several mainstream state-of-the-art (SOTA) methods across both the segmentation and classification (diagnostic) tasks. In the segmentation task, as shown in Table 8 and Figure 10a, our model exhibited outstanding performance. Compared to methods such as UNet, DynUNet, AttentionUNet, and Transformer-based models like UNETR and SwinUNETR, our model achieved the best results across all key segmentation metrics. Specifically, our model attained a Dice coefficient of 71.91± 8.50% and an IOU of 56.81 ± 10.35%, significantly surpassing all compared methods. Furthermore, its 95%HD of 9.56 ± 3.80 mm was the lowest among all methods, indicating that the segmentation boundaries generated by our model are substantially more precise. It is noteworthy that while achieving this optimal performance, our model’s parameter count (15.9 M) was significantly lower than that of larger models such as UNETR (336.4 M), demonstrating superior efficiency. The qualitative evaluation results in Figure 11 visually confirm that the segmentation mask generated by our model (red contour) exhibits the highest conformity to the ground truth, and the predictions are the smoothest and the most complete, whereas other methods suffer from fragmentation or under-segmentation to varying degrees.

In the classification (diagnostic) task, we conducted a comprehensive evaluation against both a traditional radiomics baseline model and state-of-the-art deep learning models, as illustrated in Table 9 and Figure 10b. First, to establish a quantitative baseline, we implemented a logistic regression (LR) model based on multimodal radiomics. Specifically, we extracted high-dimensional radiomic feature vectors from both the UWF images and OCT B-scans. These vectors were concatenated to form a fused feature representation, which was then used to train the LR classifier. This radiomics-based approach achieved an AUC of 71.41% and an accuracy of 71.88%. However, our proposed DME-RWKV model demonstrated a dominant advantage over this radiomics baseline model, significantly boosting the AUC to 84.36% and accuracy to 80.44%. This substantial performance margin (approx. 13% in AUC) indicates that while radiomic features can capture texture and intensity patterns, the end-to-end deep learning framework is superior in modeling complex, non-linear semantic interactions and global–local dependencies. Furthermore, compared to classical deep learning networks such as ResNet-50, DenseNet-121, SENet-50, and ViT-12, our model consistently ranked first across all evaluation metrics.

## 4. Discussion

This study is centered around the critical role of vitreomacular interface abnormalities, particularly ERM, in predicting anti-VEGF efficacy in DME. We propose a novel VRWKV architecture (DME-RWKV) that integrates ERM spatial information. The model not only jointly predicts anti-VEGF efficacy and performs OCT biomarker segmentation, but also incorporates a CAL module to accurately locate key regions. Combined with a curriculum learning strategy and GC loss, it significantly optimizes the detection of small lesions. Additionally, we developed an OCT + UWF multimodal input channel to simulate clinical decision-making processes, further enhancing prediction accuracy and interpretability, thereby providing stronger support for personalized treatment. Experimental results demonstrate that this model outperforms existing SOTA methods in both diagnostic and segmentation tasks, validating its effectiveness and clinical application potential in AI modeling guided by core clinical issues.

This study proposes a novel modeling strategy that integrates clinical priors with deep learning. In particular, we incorporated 50 quantitative imaging indicators (1 manual OCT measurement and 49 automatically extracted from UWF), integrated with end-to-end feature learning. This approach not only preserves the learning capacity of deep learning models but also explicitly integrates clinical knowledge, thus enhancing both prediction accuracy and interpretability, with a specific focus on modeling ERM-related spatial information. The framework possesses extensibility in that future incorporation of new critical imaging features can be accomplished directly without altering the underlying architecture. This allows the AI model to dynamically assimilate emerging domain knowledge and maintain consistency with clinical rationale, thereby mitigating the “black-box” nature of the model and reinforcing the central role of clinicians in the model development cycle. Experimental results demonstrate that this method outperforms existing SOTA models in both diagnostic and segmentation tasks, validating the potential of clinical-problem-driven AI modeling.

The proposed DME-RWKV model utilizes dual-channel input (OCT and UWF) and incorporates a CAL module to explicitly model the spatial features of ERM. Coupled with a curriculum learning strategy, this design strengthens the model’s recognition capability for the vitreoretinal interface. This architecture not only mimics the comprehensive assessment process used by clinicians for judging treatment efficacy—considering both local (macular microstructure) and global (retinal vascular pathology) features—but also addresses a critical limitation in previous studies. Prior research was often restricted to single-modality inputs, mostly focused on qualitative descriptions or simple statistical analyses, and lacked systematic modeling of the condition’s spatial distribution and pathological characteristics [2,5,12,32]. Our research is thus able to better delineate the potential impact of ERM on macular morphology and treatment response, yielding prediction results that are more consistent with clinical pathological rationale, and highlighting the significant value of fusing multimodal data with structured prior knowledge.

The curriculum learning strategy in this study not only simulates the stepwise diagnostic reasoning process of clinicians during training but also reflects clinical integrated decision-making logic at the multimodal data fusion level. Specifically, training samples are first categorized into three difficulty levels—easy, moderate, and difficult—based on the signal strength, area size, and image quality of the ROI regions. This approach introduces more complex cases gradually, allowing the model to first learn typical features that are easy to recognize and then progressively master more complex lesion patterns, thus enhancing convergence stability and generalization capability. Moreover, the model integrates OCT and UWF information at the input layer to simulate the comprehensive evaluation process of clinicians, who consider both local macular structures and global retinal lesions when making decisions. This integration helps to improve prediction accuracy and interpretability. The “learning process + multimodal input” dual simulation not only strengthens the model’s robustness but also provides new insights into the transferability of AI in complex clinical scenarios.

DME is a characteristic pathological manifestation that affects the macula in diabetic retinopathy and is a major cause of vision impairment in diabetic patients. The changes in other parts of the retina in diabetic retinopathy are also significant for clinical diagnosis and treatment guidance. The GCA feature fusion module significantly enhances cross-scale information interaction. Unlike traditional encoder–decoder architectures that simply concatenate or sum features from different resolutions, the GCA module models long-range dependencies and introduces a global semantic weighting mechanism. This effectively improves the discriminability of features when they are fused, allowing the network to maintain high-resolution local structures while capturing rich contextual information. This is particularly important for OCT images, as lesions can manifest as large retinal layer changes or be present only in small, localized regions.

In clinical interpretation of OCT images, clinicians do not focus on every region of the image equally but rely on pathological cues to progressively narrow their attention to critical structures. This “causal chain “ reasoning is central to maintaining diagnostic consistency. To align with this process, we designed a CAL module, which introduces causal constraints during feature extraction, aligning attention distribution with clinical diagnostic reasoning and reducing the model’s response to irrelevant background noise. This mechanism improves the model’s sensitivity to key structures, particularly in cases where lesion boundaries are vague or lesion signals are weak, thus enhancing prediction stability and reliability. The quality of the attention maps optimized by CAL significantly improves, increasing the accuracy of identifying key features associated with DME by 10%. This method is not only applicable to DME but can also extend to other ophthalmic diseases, broadening its potential application in the field of ophthalmology.

In OCT images, small and elongated lesions (such as focal edema or exudative bands) significantly affect the treatment prognosis. These lesions are closely monitored by clinicians, as their location, number, and activity directly influence immediate treatment outcomes. Traditional segmentation methods (e.g., Dice Loss) often fail to maintain structural continuity when processing such lesions. The introduction of the GC loss function effectively addresses this challenge by improving segmentation continuity and structural integrity. The accuracy of segmenting small lesions is closely related to patient prognosis and can provide crucial data for early intervention, guiding treatment adjustments.

Compared to existing SOTA methods, DME-RWKV achieves significant performance advantages in both diagnostic and segmentation tasks, particularly in small lesion detection and cross-scale feature fusion. As summarized in Table 10, this superior performance can be attributed to three key qualitative advantages of our framework over existing methods: (1) Efficiency–accuracy balance: unlike CNNs, which lack global context, and Transformers, which are computationally heavy, our RWKV-based encoder captures long-range dependencies with linear complexity, enabling efficient processing of high-resolution OCT images. (2) Structural integrity for micro-lesions: the introduction of the GC loss function explicitly addresses the topological breakage often seen in CNN-based segmentation of small lesions (e.g., IRC, SRF), ensuring continuous and precise boundaries. (3) Clinical interpretability: By integrating the CAL module and a multimodal (OCT + UWF) curriculum learning strategy, the model mimics clinical reasoning. This not only improves robustness against noise but also provides causally relevant attention maps, making the “black-box” decision process transparent to clinicians. These combined advantages suggest that our comprehensive framework better meets the dual clinical demands for precision and stability, holding strong potential for broader application and value.

Despite the encouraging results, this study has several limitations that need to be addressed. First, the retrospective study design and the fact that all data were derived from a single tertiary hospital may introduce selection bias and limit the generalizability of our model. The patient demographics, treatment protocols, and imaging equipment characteristics at our center may not fully represent the broader and more diverse populations and clinical settings encountered in real-world practice. Therefore, the performance of DME-RWKV needs external validation at multiple geographically dispersed centers to confirm its robustness and general applicability. A larger-scale prospective observational trial using standardized imaging protocols is necessary to confirm and extend these findings. Secondly, the sample included in this study exhibited a class imbalance, with the non-responsive group having a larger number than the responsive group. Although this reflects the objective description of real-world clinical outcomes, it may introduce certain biases. Future studies should aim to further eliminate intergroup differences to ensure the rigor of the research. Finally, although the model incorporates interpretability modules (such as CAL), its ultimate clinical utility and impact on actual treatment decisions still require prospective evaluation within clinical workflows. Therefore, future work should focus on conducting multi-center external validation and prospective trials to assess the model’s efficacy in improving patient outcomes and optimizing clinical decision-making in DME management.

## 5. Conclusions

In conclusion, this study presents a pioneering multi-task analysis framework for OCT images and makes three distinct contributions to the field of ophthalmic AI. First, in terms of architectural innovation, we propose the DME-RWKV, which effectively balances global context modeling with computational efficiency. Unlike traditional CNNs restricted by local receptive fields or Transformers burdened by quadratic complexity, our introduction of the linear-complexity RWKV mechanism allows for efficient processing of high-resolution retinal images. Second, regarding methodological advancement, we explicitly integrate clinical reasoning into deep learning. By designing the CAL module and a curriculum learning strategy, the model mimics the “stepwise” and “evidence-based” diagnostic process of clinicians, significantly enhancing the interpretability and robustness of the “black-box” decision process. Third, addressing the specific challenge of micro-lesion detection, we introduce a novel GC loss. This contribution overcomes the limitations of pixel-wise loss functions (e.g., Dice Loss) by enforcing topological structural integrity, thereby resolving the fragmentation issue in segmenting small lesions like IRC and SRF.

Collectively, these contributions establish a robust system that does not rely solely on overall image patterns but integrates anatomical priors and multimodal data (OCT and UWF) to explicitly characterize vitreomacular interface abnormalities. Future work could further achieve “joint operations” by fusing multimodal imaging (e.g., OCT angiography, fundus photography), functional assessments, and clinical variables to develop a model adaptable across different disease types, which would enhance the developed AI system’s overall understanding and generalization ability for complex ocular conditions.

## Figures and Tables

**Table 1:** Baseline patient characteristics.

		Total Mean ± SD/N (%)	Responsive Group Mean ± SD/N (%)	Non-Responsive Group Mean ± SD/N (%)	*p* Value
Age		63.54 ± 12.01	62.1 ± 12.86	64.53 ± 11.33	0.164
Gender					0.010
	Female	153 (41.24%)	73 (49.66%)	80 (35.71%))	
	Male	218 (58.76%)	74 (50.34%)	144 (64.29%)	
ERM		93 (25.06%)	43 (29.25%)	49 (21.88%)	0.137
CMT		428.46 ± 220.08	530.76 ± 230.57	361.33 ± 184.76	<0.001
IOP		16.24 ± 2	16.2 ± 1.93	16.27 ± 2.04	0.726
BCVA		0.23 ± 0.19	0.19 ± 0.18	0.26 ± 0.2	0.003

Numerical data: Mann–Whitney U test; categorical data: Chi-square test.

**Table 2:** Quantitative comparison of single-modal and multimodal (OCT + UWF) inputs.

Encoder Block	Module	Modal	Dice (%)	IOU (%)	AUC (%)	ACC (%)
HPA + RWKV	CAL + GCA	OCT	62.26 ± 10.46	46.05 ± 11.40	75.26 (71.45–78.85) **	71.49 (68.38–74.61)
		UWF	-	-	75.99 (72.62–79.42) **	70.63 (67.13–74.30)
		OCT + UWF	**67.32 ± 8.35**	**51.33 ± 9.73**	**79.69 (69.41–89.51)**	**79.61(71.62–87.84)**

Note: DeLong’s test was performed to compare the AUC values. ** indicates *p* < 0.001. Bold text represents the best result.

**Table 3:** Quantitative evaluation of segmentation performance across different modules of DME-RWKV.

Encoder Block	Module	Dice (%)	IOU (%)	95%HD	Params. (M)
RWKV (4 Stages)	-	61.82 ± 13.35	46.03 ± 13.81	12.74 ± 5.47	24.2
HPA (4 Stages)		61.08 ± 12.13	45.09 ± 13.34	13.69 ± 4.59	**5.7**
HPA + RWKV		62.96 ± 12.08	47.04 ± 12.88	13.26 ± 4.82	7.6
HPA + RWKV	CAL	63.84 ± 12.68	48.19 ± 14.56	12.67 ± 4.80	9.4
HPA + RWKV	GCA	65.77 ± 11.62	50.12 ± 13.36	11.55 ± 4.31	10.3
HPA + RWKV	CAL + GCA	**67.32 ± 8.35**	**51.33 ± 9.73**	**11.91 ± 4.27**	15.9

Note: Bold text represents the best result.

**Table 4:** Quantitative evaluation of DME prediction performance across different modules of DME-RWKV.

Encoder Block	Module	AUC (%)	Delong’s Test	ACC (%)	SEN (%)	SPE (%)
RWKV (4 Stages)	-	73.35 (60.65–84.91)	0.0002 **	75.21 (66.22–85.14)	83.97 (64.52–96.67)	66.52 (50.00–85.71)
HPA (4 Stages)		70.52 (58.22–81.87)	0.0063 *	70.69 (60.81–79.73)	86.37 (52.77–92.41)	55.26 (33.33–86.84)
HPA + RWKV		74.55 (62.53–85.83)	0.0039 *	73.60 (63.51–82.43)	69.56 (43.17–91.89)	77.68 (55.17–94.87)
HPA + RWKV	CAL	76.09 (63.99–86.52)	0.0012 *	73.65 (64.83–82.43)	73.29 (41.03–97.44)	73.93 (43.24–97.62)
HPA + RWKV	GCA	75.58 (64.13–86.03)	0.0021 *	75.21 (66.22–85.14)	78.33 (55.17–93.55)	72.17 (54.55–90.25)
HPA + RWKV	CAL + GCA	**79.69 (69.41–89.51)**	**-**	**79.61 (71.62–87.84)**	**83.41 (66.67–94.24)**	75.83 (52.50–90.91)

Note: DeLong’s test was performed to compare the AUC values. * indicates 0.001 < *p* < 0.05, and ** indicates *p* < 0.001. Bold text represents the best result.

**Table 5:** Quantitative evaluation of DME-RWKV with and without joint prediction and segmentation.

Task	Dice (%)	IOU (%)	AUC (%)	ACC (%)	SEN (%)	SPE (%)
Segmentation	65.36 ± 10.47	49.45 ± 12.02	-	-	-	-
Prediction	-	-	77.23 (65.71–87.62) *	74.69 (64.86–83.78)	69.87 (38.88–94.60)	**79.49 (53.83–91.49)**
**Union**	**67.32 ± 8.35**	**51.33 ± 9.73**	**79.69 (69.41–89.51)**	**79.61 (71.62–87.84)**	**83.41 (66.67–94.24)**	75.83 (52.50–90.91)

Note: DeLong’s test was performed to compare the AUC values. * indicates 0.001 < *p* < 0.05. Bold text represents the best result.

**Table 6:** Quantitative results of the ablation study on the loss function.

Loss	*s*	Dice (%)	IOU (%)	AUC (%)	ACC (%)	SEN (%)	SPE (%)
DCE	5	67.32 ± 8.35	51.33 ± 9.73	79.69 (69.41–89.51) *	79.61 (71.62–87.84)	83.41 (66.67–94.24)	75.83 (52.50–90.91)
DCE + clDice	5	69.74 ± 9.80	54.38 ± 11.62	80.13 (68.88–90.70) *	78.56 (68.92–87.84)	72.06 (53.33–91.43)	85.12 (64.28–97.50)
**DCE + GC**	**5**	**70.69 ± 8.72**	**55.33 ± 10.21**	**81.98 (71.65–90.48)**	**78.41 (70.27–86.49)**	**74.70(45.95–92.86)**	**82.24 (62.86–94.13)**
	3	67.22 ± 9.00	51.31 ± 10.34	77.01 (71.41–82.51) *	73.05 (67.97–77.74)	80.55 (54.92–91.18)	65.59 (51.59–87.14)
	7	69.99 ± 9.12	54.64 ± 11.94	77.35 (71.21–82.87) *	72.66 (67.58–77.73)	74.31 (59.70–93.02)	71.01 (49.59–84.79)

Note: DeLong’s test was performed to compare the AUC values. * indicates 0.001 < *p* < 0.05. Bold text represents the best result.

**Table 7:** Quantitative evaluation of DME-RWKV with curriculum learning.

Stage	Dice (%)	IOU (%)	AUC (%)	ACC (%)	SEN (%)	SPE (%)
I	66.47 ± 11.28	50.90 ± 13.74	76.22 (65.17–86.31) **	74.75 (64.86–83.78)	81.98 (59.42–97.44)	67.69 (46.34–86.68)
II	68.92 ± 10.81	53.59 ± 12.62	81.61 (71.54–89.89) *	76.83 (68.89–85.14)	80.24 (45.94–94.41)	73.66 (47.06–96.77)
III	**71.91 ± 8.50**	**56.81 ± 10.35**	**84.36(75.09–92.62)**	**80.44(71.62–89.19)**	76.37 (57.89–96.67)	**84.49 (60.97–97.44)**

Note: DeLong’s test was performed to compare the AUC values. * indicates 0.001 < *p* < 0.05, and ** indicates *p* < 0.001. Bold text represents the best result.

**Table 8:** Quantitative evaluation results of comparison with other segmentation methods.

Methods	Dice (%)	IOU (%)	95% HD	Params. (M)
UNet	50.94 ± 18.48	36.12 ± 16.29	17.52 ± 6.55	**1.6**
DynUNet	55.34 ± 11.92	39.21 ± 11.96	15.47 ± 4.58	30.2
AttentionUNet	57.48 ± 16.91	42.24 ± 16.79	15.24 ± 6.30	7.5
UNETR	58.67 ± 15.21	43.11 ± 15.44	14.24 ± 4.87	336.4
SwinUNETR	56.56 ± 12.67	40.46 ± 12.13	14.95 ± 4.86	29.5
RWKV-UNet	60.66 ± 13.71	44.93 ± 14.69	13.76 ± 5.45	24.2
Ours	**71.91 ± 8.50**	**56.81 ± 10.35**	**9.56 ± 3.80**	15.9

Note: Bold text represents the best result.

**Table 9:** Quantitative evaluation results of comparison with other prediction/classification methods.

Methods	AUC (%)	ACC (%)	SEN (%)	SPE (%)	F1 (%)	PRE (%)
LR	71.41 (59.40–82.77)	71.88 (62.50–81.25) **	69.69 (40.48–95.12)	74.11 (40.62–95.24)	70.46 (54.84–81.93)	73.87 (56.36–90.92)
ResNet-50	65.90 (52.64–78.36)	67.84 (58.11–78.38) **	68.04 (25.57–90.91)	67.70 (42.86–88.51)	66.48 (40.00–80.44)	70.51 (53.06–86.41)
DenseNet-121	70.80 (58.54–82.52)	70.36 (60.81–79.73) **	64.86 (39.47–89.81)	75.88 (32.43–97.06)	67.66 (52.83–80.52)	75.39 (54.55–95.45)
SENet-50	65.95 (54.05–78.24)	67.45 (58.11–77.03) **	69.40 (39.47–96.87)	65.86 (29.27–91.67)	67.11 (49.98–78.43)	68.51 (50.88–86.96)
ViT-12	72.35 (60.32–82.52)	72.18 (62.16–81.08) **	90.39 (58.82–97.45)	53.84 (30.30–84.09)	76.19 (64.51–84.91)	67.02 (52.46–82.24)
Ours	**84.36 (75.09–92.62)**	**80.44 (71.62–89.19)**	76.37 (57.89–96.67)	**84.49 (60.97–97.44)**	**79.24 (68.00–88.61)**	**83.99 (65.79–96.67)**

Note: DeLong’s test was performed to compare the AUC values. ** indicates *p* < 0.001. Bold text represents the best result.

**Table 10:** Qualitative comparison of the proposed DME-RWKV with SOTA methods.

Feature/Capability	CNN-Based Methods (e.g., ResNet, UNet)	Transformer-Based Methods (e.g., ViT, UNETR)	Proposed DME-RWKV
Global Context Modeling	Limited (Local Receptive Fields)	Excellent (Self-Attention)	Excellent (Linear Attention via RWKV)
Computational Efficiency	High	Low (Quadratic Complexity)	High (Linear Complexity)
Small Lesion Detection	Prone to Fragmentation (Dice Loss Limitation)	Moderate	Superior (Enhanced by GC Loss)
Interpretability	Low (“Black-box”)	Moderate (Attention Maps)	High (Causal Attention/CAL Module)
Multimodal Integration	Typically Single-modal	Typically Single-modal	OCT + UWF Dual-modal
Training Strategy	Standard	Standard	Curriculum Learning (Easy-to-Hard)

## Data Availability

For ethical reasons, the data used in this study should be provided upon request to the corresponding author.

## Abbreviations

The following abbreviations are used in this manuscript:

AI	Artificial intelligence
BCVA	Best-corrected visual acuity
CAL	Causal attention learning
CMT	Central macular thickness
CNNs	Convolutional neural networks
DME	Diabetic macular edema
ERM	Epiretinal membrane
GC	Global completion
GCA	Global-context attention
HPA	Hadamard-projected attention
IOP	Intraocular pressure
IRC	Intraretinal cysts
OCT	Optical coherence tomography
RNNs	Recurrent neural networks
ROC	Receiver operating characteristic
ROI	Region of interest
RWKV	Receptance weighted key value
SOTA	State of the art
SRF	Subretinal fluid
VEGF	Vascular endothelial growth factor
ViTs	Vision transformers
VRWKV	Vision-RWKV
UWF	Ultra-widefield
